# Thermal stability of idealized folded carbyne loops

**DOI:** 10.1186/1556-276X-8-490

**Published:** 2013-11-20

**Authors:** Steven W Cranford

**Affiliations:** 1 Laboratory of Nanotechnology in Civil Engineering, Department of Civil and Environmental Engineering, Northeastern University, Boston, MA 02115, USA

**Keywords:** Carbyne, Molecular dynamics, Unfolding, Adhesion, Torsion, Curvature, Stability

## Abstract

Self-unfolding items provide a practical convenience, wherein ring-like frames are contorted into a state of equilibrium and subsequently  pop up’ or deploy when perturbed from a folded structure. Can the same process be exploited at the molecular scale? At the limiting scale is a closed chain of single atoms, used here to investigate the limits of stability of such folded ring structures via full atomistic molecular dynamics. Carbyne is a one-dimensional carbon allotrope composed of *sp*-hybridized carbon atoms. Here, we explore the stability of idealized carbyne loops as a function of chain length, curvature, and temperature, and delineate an effective phase diagram between folded and unfolded states. We find that while overall curvature is reduced, in addition to torsional and self-adhesive energy barriers, a local increase in curvature results in the largest impedance to unfolding.

## Background

A clever trick by product designers is self-unfolding structures such as collapsible laundry hampers and  pop-up’ tents. These ingenious designs involve a continuous ring structure that  unfolds’ to a larger configuration. Similar mechanisms have been proposed for systems ranging from stretchable electronics [1] to polymer membranes [2,3] and hollow shell structures [4]. Here, we focus on the smallest possible unfolding system - a closed chain of carbon atoms - to investigate the limits of stability at the atomistic scale. Insights from such structures can then be applied to more complex macromolecular systems, such as responsive polymer [5,6] or protein-based materials [7-10].

**Figure 1:**
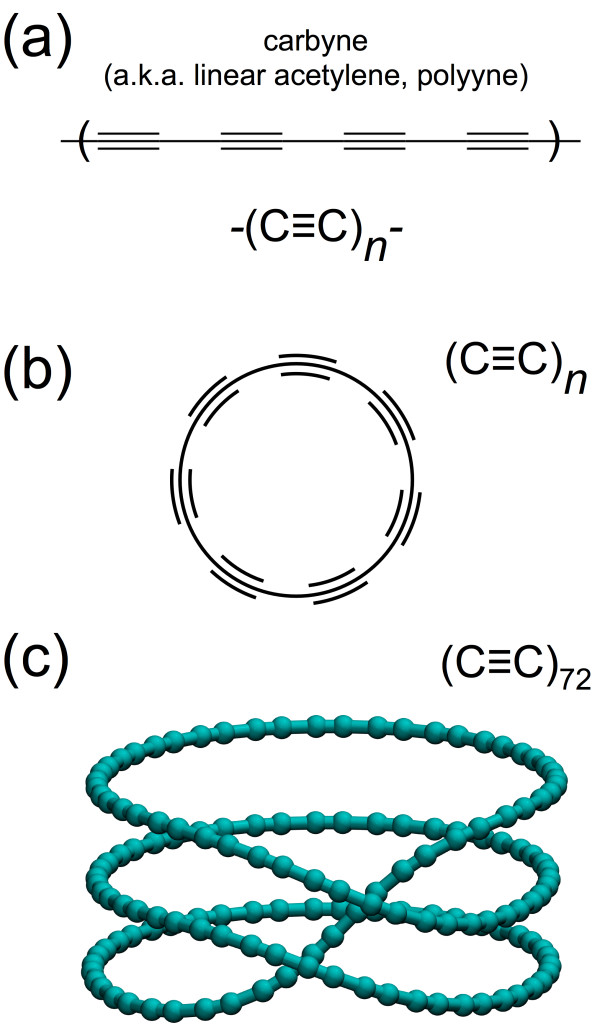
**Three-loop carbyne model and simulation. (a)** Molecular structure of carbyne, a one-dimensional carbon allotrope composed of *sp*-hybridized carbon atoms, consisting of alternating single-triple bonds. While chains of carbyne can be experimentally synthesized, they typically require heavy end-groups for stability [12,19]. **(b)** A theoretical carbyne  loop’, circumventing the need for stabilizing end-groups by bonding the carbyne chain to itself. **(c)** Example molecular model of a folded carbyne loop in a stable three-ring configuration, with imposed overcurvature of three [68], similar to self-unfolding laundry hampers.

A simple molecular system capable of folding into a simple ring structure while maintaining atomistic fidelity and behavior is desired. As such, a model system is constructed using carbyne - a one-dimensional carbon allotrope consisting of either a cumulative double-bond structure (cumulene) or alternating single and triple bonds (polyyne) [11,12]; the polyyne structure is depicted in Figure 1a. This 1D carbon structure has caused recent interest due to its novel electron transport and the prospect of being components in atomistic scale circuits [13,14], as well as recent synthesis of long chains [15-19]. Previous first-principle- and molecular dynamics (MD)-based studies [20-23] have characterized molecular mechanics (e.g., zero or near-zero temperatures) properties of isolated carbyne chains (e.g., in a vacuum). Considered here is a system of isolated closed-loop carbyne (Figure 1b) to explore the stability of a folded three-loop geometry (Figure 1c).

In simplest terms, additional elastic strain energy due to curvature triggers unfolding from the three-loop configuration. However, to completely unfold from an initial coiled state at the molecular scale, both torsional and self-adhesive energetic barriers must be overcome, resulting in a range of stable conditions, depending on initial curvature (*κ*) and temperature (*T*). In terms of coiled, looped, or otherwise convoluted geometries (such as knotted molecular structures [24,25]), a mono-atomistic linear structure provides the simplest and most fundamental platform to explore stability and unfolding. Other systems, such as convoluted protein structures or DNA, would be more complex to analyze (due to kinetic hindrance of side-chain interactions, for example), but similar looped structures exist [26-28] and are also dictated by a balance of thermal and mechanical contributions [29-31].

While linear carbon chains have been experimentally attained, such a closed carbyne has yet to be synthesized. However, recent developments of carbon materials such as annulenes [32-34] and extended porphyrins [35] suggest that carbon may allow such  atomistic control’ and design of such molecular structures. Similar folded/looped atomistic structures include molecular knots [36,37], foldamers [38,39], and cyclic heterostructures [39-42]. The use of homogeneous carbon eliminates the effects of more complex structures (such as torsional rigidity or steric interactions). However, while carbyne is used here as an idealized model system, the general behavior can serve as an analog to such systems and reflect the dynamics at a molecular scale.

## Methods

Full atomistic simulations are implemented using classical MD, utilizing the first-principle-based ReaxFF potential [43,44], known to provide an accurate account of the chemical/mechanical behavior of carbon nanostructures [21,45-49]. Due to a bond order-based formulation, ReaxFF can reflect the bond hybridization of the polyyne structure of carbyne, as well as the effect of other valence terms (angle and torsion), without explicit parameterization [45]. It is noted that at such a scale, electron behavior may play a critical role. For example, a previous study demonstrated that in linear carbon chains, a local perturbation through the displacement of a single atom creates atomic force and charge density Friedel-like oscillations [50]. Other electron-dependent effects may include Jahn-Teller distortions [51] or Möbius topologies [52,53]. While such complex behavior is incapable of being replicated by MD potentials, it is deemed sufficient for the current scope of length and temperature effects on unfolding. A time step is chosen to be on the order of a fraction of femtoseconds (0.1 × 10^-15^ s) to ensure the stability and reflect the high vibrational frequency of the acetylene groups of carbyne. All simulations are subject to a canonical (NVT) ensemble, with varying prescribed temperature (10 to 800 K), performed using the massively paralyzed modeling code LAMMPS (http://lammps.sandia.gov/) [54].

**Figure 2:**
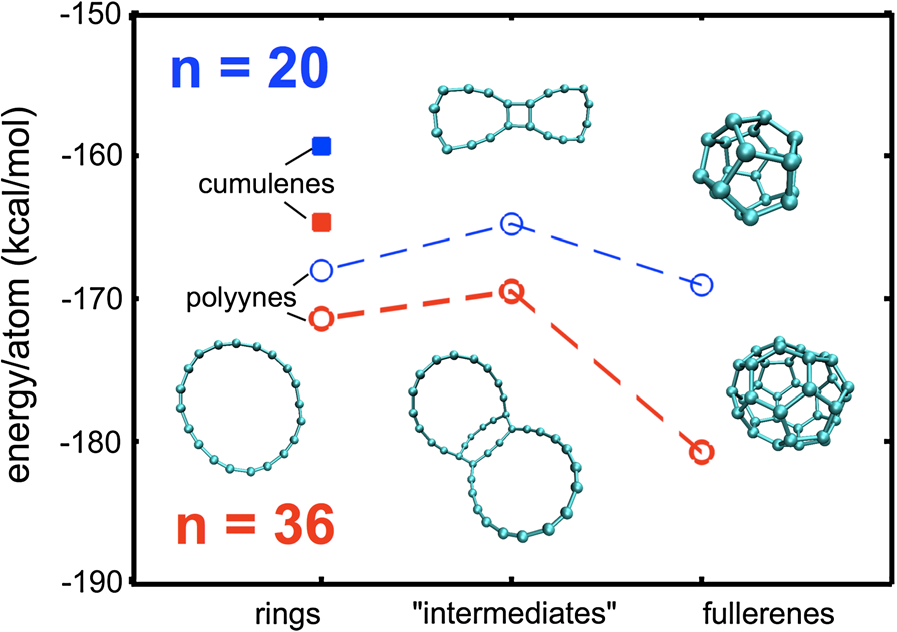
**Relative molecular stability.** Carbyne rings have been proposed as a transitional form of carbon during the synthesis of fullerenes [60-63]. Other intermediate forms occurring with chain self-adhesion may form (e.g., so-called bow tie structures). To assess the stability of the rings during folding/three-loop configuration, the atomistic energy of cumulene rings and example  intermediate’ structures was assessed with *n* = 20 (top, blue) and *n* = 36 (bottom, red) carbon atoms. We see that, aside from the fullerenes, the closed-loop ring polyyne carbyne structures are more energetically favorable (lower energy) than the intermediates depicted, suggesting a relative stability for the equilibrium simulations undertaken.

As carbyne has been stated to take either a cumulene (=C = C=) or a polyyne form (-C ≡ C-), small test structures (rings with *n* = 20 and *n* = 36) were minimized using ReaxFF to check the relative energetic stability of each structure (Figure 2). Cumulene has been reported to undergo a Peierls transition [55] into a polyyne form; our simulations confirm this instability, showing an energy difference in the order of 6.7 to 8.6 kcal mol^-1^ atom^-1^ in favor of the looped polyyne, in agreement with previous studies [55,56]. Moreover, beyond minimization, when nominal temperature was added to the ring structures, the cumulene rings transitioned to a triple-single bond pattern, potentially due to the strain associated with the imposed curvature, which can facilitate the transition [57]. As the focus here is variation in temperature, only the polyyne configuration is stable throughout the range of temperatures used. Thus, all carbyne ring structures considered are reflective of polyyne structures. Initial three-loop systems are constructed with 54, 72, 90, 108, 126, 144, 162, and 180 carbon atoms, with associated ideal radii of approximately 4 to 13 Å. The three-loop fold pattern imposed is meant to maintain a near-constant curvature across the total molecular length.

In terms of the ring structure, while linear carbyne chains have been shown to be stable [19,58], imposing a closed-loop geometry may be energetically unfavorable. To directly assess the stability of looped carbyne here, a linear chain was equilibrated to determine the difference in atomistic energy in comparison with the 54-atom looped structure, resulting in a nominal difference of 0.02 eV atom^-1^ and suggesting structural stability. For comparison, the energy difference between flat graphene and a fullerene is in the order of 0.2 eV atom^-1^[59], while the cohesive energy of carbyne has been found to be in the order of 6.99 [56] to 8.19 eV atom^-1^[50], in close agreement with the value of 7.4 eV atom^-1^ calculated here at a finite temperature of 300 K.

We also wish to assess the stability in comparison with other non-carbyne molecular configurations. Empirically, similar ring-like structures with as few as 20 carbon atoms have been observed in the synthesis of fullerenes [60], as well as many intermediate bonded chain forms (e.g., so-called bow tie structures or cycloadducts) [60-63]. To explore whether such intermediate forms may be energetically favorable, simple trial structures were equilibrated to assess the potential energy (also depicted in Figure 2), indicating that the looped/ring structure is more favorable than other intermediate forms. As depicted in Figure 2, the most energetically favorable structures are the fullerenes, with rings more favorable than that of the  intermediate’ structures (in agreement with previous studies [61]; absolute relative energy differences would be dependent on the MD potential used). Increasing the bending energy (through folding) could increase the energy such that a transition may occur. That being said, the modeled structure not being the most energetically favorable does not imply that it cannot exist. Such an argument would indicate that fullerenes themselves should not exist, yet C_20_ fullerenes, bowls, and rings have been observed [60]. Less favorable intermediate structures are proposed pathways to fullerene synthesis [62,63] and can exhibit interesting properties or result in the synthesis of unique structures [64]. The focus here only involves the stability of a presumed folded structure.

The looped structures are equilibrated at a nominal temperature (10 K) and then subjected to temperature increase to a target temperature (with a rate of approximately 0.001 K fs^-1^) over 10 ps. As the molecular structures are isolated in a vacuum, the use of temperature as a variable is a direct measure of the kinetic energy of the atoms, independent of any insulating or damping effects an explicit solvent may contribute. Once the target temperature is reached, constant temperature is maintained, and the system is allowed to freely evolve for up to 0.1 ns to assess the stability of the configuration (test trials up to 5.0 ns were also ran to ensure equilibrium; in all cases, if unfolding was initiated, it occurred at a timescale less than 0.1 ns). The critical temperature of unfolding is then determined for each structure. Since the process is stochastic across the chain and the temperature is an ensemble average, the designated unfolding temperature only approximates the magnitude of energy required to trigger unfolding, and thus a range of critical temperatures emerges for the structures across multiple simulations. While the temperature variation was used to induce unfolding, of note is that the carbyne chains do not begin to disassociate until temperatures exceed approximately 3,500 K regardless of size (and a loss of any definitive curvature), defining an accessible temperature range for the ring structures.

## Results and discussion

### Root mean square deviation

**Figure 3:**
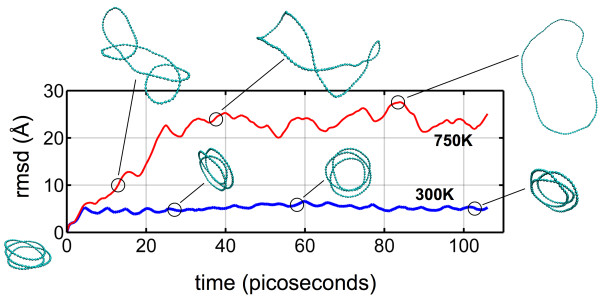
**Simulation snapshots and root mean square displacement (or rmsd; see Equation** 1**) trajectories.** Structures for *n* = 144 during low- and high-temperature simulations. For low temperature (300 K, bottom), the folded three-loop structure remains stable and is an equilibrated state (indicated by the relatively constant RMSD). Increasing the temperature (750 K, top) induces unfolding, after which the unfolded structure equilibrates (larger variation in RMSD due to the oscillations induced by the momentum of unfolding).

Example snapshots of an unfolding loop are given in Figure 3, along with the associated root mean square deviation (RMSD) plot. The RMSD is defined as the spatial difference between two molecular structures:

(1) $$\text{RMSD}=\sqrt{\frac{1}{N}\sum_{i=1}^{N}{\left(r_{i}\left(t\right)-r_{0,i}\right)}^{2}}$$

where *N* denotes the number of atoms, *r*(*t*) denotes the position of each atom in the structure at time *t*, and *r*_0_ denotes the positions for the initial three-loop structure. A plateau of RMSD values indicates a locally stable structure and relative equilibrium. Due to the drastic structural transition between folded and unfolded states, RMSD can be used as a consistent metric to assess the stability of the global conformation [65,66]. The results indicate that unfolding occurs on a fast timescale on the order of tens of picoseconds once initiated. For comparison, such timescales have been observed on local/partial unfolding events of larger protein structures [66,67].

### Adhesion and torsional barriers

A recent macroscale investigation has determined that the way these rings behave depends on a single characteristic known as overcurvature [68] or how much more curved the three-loop configuration is than a flat circle of the same circumference. Here, each structure has the same initial overcurvature (equal to three). However, at the molecular scale, where temperature and self-adhesion effects are on the same energetic scale as strain energy, the relationship between curvature and stability is more complex. Indeed, due to the imposed overcurvature of the three-loop conformation, it could be anticipated that a relaxation of bending strain energy results in the necessary energy to unfold, assuming that the energy is sufficient to overcome the energy barrier due to adhesion and/or torsion (a full twist/rotation is necessary to unfold a looped chain).

**Figure 4:**
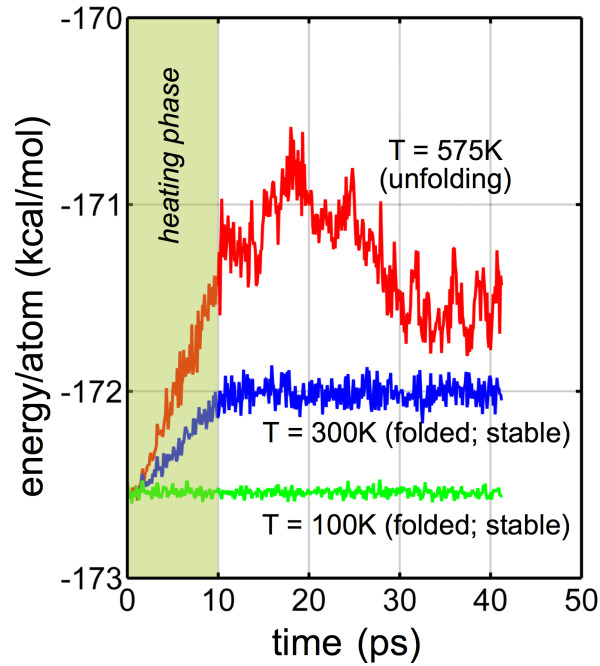
**Representative potential energy evolution for various temperatures (*T*** **= 100, 300, and 575 K) for *n*** **= 126.** Initial heating phase (10 ps) increases energy due to temperature until either the structure remains in a folded, stable equilibrium (100, 300 K) or unfolding is triggered (575 K). Unfolding at the critical temperature is characterized by a drop in energy due to the release of bending strain energy and global increase in curvature. Here, the critical unfolding energy barrier is approximately 217 kcal mol^-1^.

**Figure 5:**
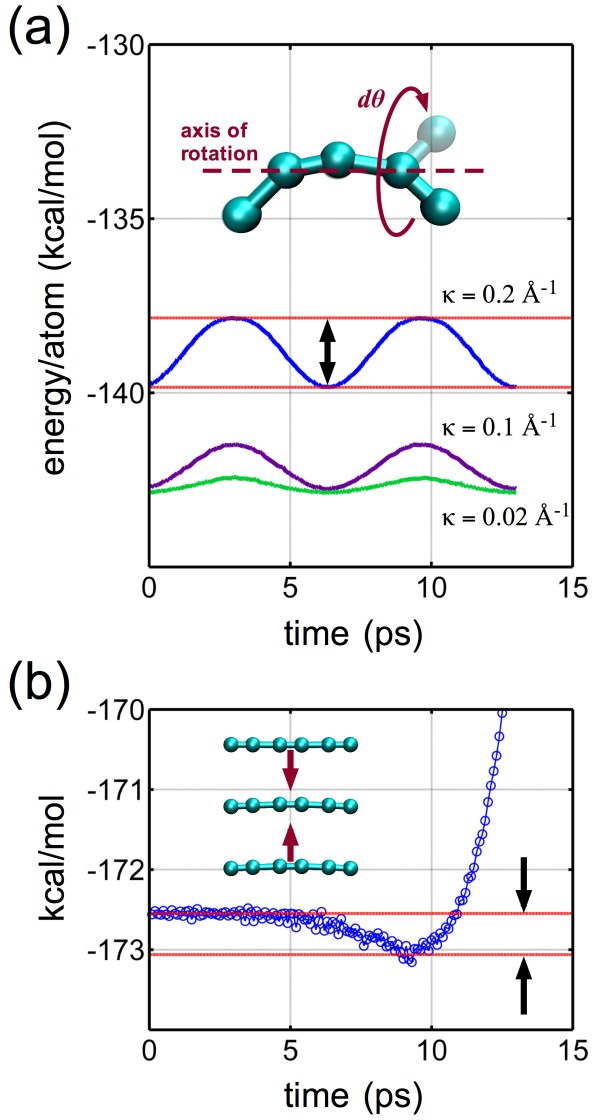
**Determining torsional and self-adhesion energy barriers. (a)** Torsion: A simple five-atom carbyne system with an imposed curvature (*κ* = 0.016 to 0.395 Å^-1^, inset *κ* = 0.2 Å^-1^) is subject to incremental twist while tracking the potential energy. The cyclical energy change due to a 180° twist increases with initial curvature as shown, defining the energy barrier (indicated by arrows) to untwist a carbyne chain in the looped configuration. **(b)** Adhesion: Three short six-atom carbyne chains (to reflect a three-loop adhesion case) were brought into close proximity over time to determine the interchain adhesion energy barrier, defined as the depth of the potential energy well (indicated by arrows).

Beyond the RMSD calculation, we track the associated potential energy of the carbyne system at a given temperature as it either remains stable (and in a three-loop configuration) or unfolds. Representative results are plotted in Figure 4. The given example indicates an energy barrier in the order of 200 kcal mol^-1^ (for *n* = 126 and an unfolding temperature of 575 K). For all systems (54 to 180 atoms), the energy barriers were approximately 40 kcal mol^-1^ (*n* = 54) to 400 kcal mol^-1^ (*n* = 180), indicating a clear length dependence on the unfolding energy. To explore the magnitude of the absolute energy barrier due to torsion and adhesion, small simulations to explicitly quantify the energy of each contribution were undertaken independently (Figure 5).

For torsion, involving a complete rotation of the carbyne chain about itself, the associated energy barrier would be a function of the initial curvature. A simple five-atom chain was constructed with a set of 14 initial curvatures ranging from 0.016 to 0.395 Å^-1^ and subjected to incremental twist while tracking the potential energy (representative plots are given in Figure 5a). During the simulation, one terminal atom is fixed, along with the second-to-the-last atom at the opposite end, while the adjacent terminal atom is then rotated about an axis of rotation and constant curvature maintained. The maximum energy barrier was calculated to be approximately 10 kcal mol^-1^, exhibited at large curvatures (>0.1 Å^-1^). A recent study quantified the torsional stiffness of carbyne, albeit using *ab initio* methods, a straight chain configuration, and the rotation of end-groups [56]. The reported energy barrier due to torsion ranged from approximately 0.2 to 0.6 eV, or 5 to 14 kcal mol^-1^. While the simulation approach and boundary conditions were different, the energy barrier determined here (approximately 10 kcal mol^-1^) is in the same order of magnitude and in a relatively good agreement. For adhesion, three carbyne chains were brought into contact and incrementally separated to determine the interchain adhesion energy (Figure 5b) of approximately 0.5 kcal mol^-1^ atom^-1^. For the worst case scenario (the longest chain of 180 carbons resulting in three adhered 60 carbon rings plus the highest recorded torsional barrier), we calculate a maximum energy barrier of approximately 40 kcal mol^-1^ - smaller than all but the minimum (*n* = 54) required energy increase indicated by the unfolding structures (also note that *n* = 54 unfolds with nominal kinetic energy required, at approximately *T ≈* 10 K, representing the smallest possible stable three-loop structure). This indicates an additional contribution that must be overcome to induce unfolding, and we hence turn to the analysis of curvature.

### Global and local curvature analysis

**Figure 6:**
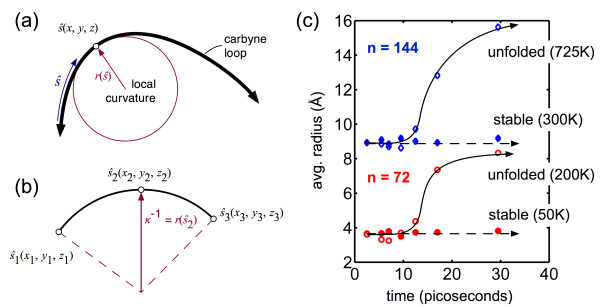
**Curvature definition and global unfolding. (a)** Defining local radius of curvature, *r*(*ŝ*), in the carbyne loop (*ŝ =* 0 to *L*), averaged to calculate the global radius of curvature and *κ*. **(b)** Schematic of coordinates used for the numerical solution to Equation 2, where each point represents adjacent carbon atoms. **(c)** Averaging the local curvatures across the molecule (here, *n* = 72 and *n* = 144) and calculating the associated radius of curvature, stable loop configurations have little change in radius at low temperatures (dashed arrows), while unfolding induced by high temperature results in a global increase in radius with respect to time (solid arrows) as anticipated (by definition, the unfolded structure will have a lower curvature).

First, we assess the evolution of curvature between stable and unfolding structures through the equation [69]

(2) $$\kappa \left(\hat{s}\right)=\frac{\sqrt{{\left(z^{''}y^{'}-y^{''}z^{'}\right)}^{2}+{\left(x^{''}z^{'}-z^{''}x^{'}\right)}^{2}+{\left(y^{''}x^{'}-x^{''}y\right)}^{2}}}{{\left(x^{'2}+y^{'2}+z^{'2}\right)}^{3/2}}$$

where the derivatives with respect to the to the chain length, *ŝ* = 0 to *L*, and can be solved numerically through the coordinates of the atoms (Figure 6a,b). Representative results are depicted in Figure 6c, indicating the average radius of curvature $\left(r_{\mathrm{avg}}=\kappa_{\mathrm{avg}}^{-1}\right)$ of the molecular loop during simulation. For stable conditions, the average radius is approximately constant (with thermal fluctuations). In contrast, temperature-induced unfolding results in a corresponding increase in radius (from 3.7 to 8.3 Å for *n* = 72 and 9.0 to 15.6 Å for *n* = 144 loops, respectively). From this *global* perspective, the loop is homogeneously *unfolding*, which would lead to a constant decrease in potential energy. The average radius of curvature, however, is insufficient to describe the more complex dynamics of unfolding. The linked and continuous looped structure impedes homogeneous relaxation of curvature; indeed, for sections of the structure to unfold, instantaneous *increase* in local curvature is observed. In effect, the relaxation of one or two loops results in the local bending increase of adjacent carbon bonds.

**Figure 7:**
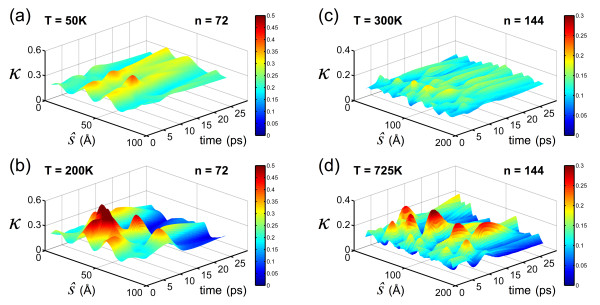
**Local curvature, *κ*(*ŝ*,*t*). (a)** Curvature across molecule for *n* = 72 at a stable low temperature (50 K). The curvature across the molecule is approximately constant (with thermal fluctuations); average, approximately 0.27 Å^-1^. **(b)** At a higher temperature (*T* = 200 K), the structure is unstable and undergoes unfolding. Unfolding induces localized increases in curvature resulting in large peaks (*к* → 0.5 Å^-1^) for sections of the molecule length. Once sufficient unfolding occurs, the structure approaches a homogeneous, unfolded state (*κ* ≈ 0.12 Å^-1^). **(c)** Curvature across molecule for *n* = 144 at a stable low temperature (300 K). Again, the curvature across the molecule is approximately constant; average, approximately 0.11 Å^-1^. **(d)** At a higher temperature (*T* = 725 K), the longer structure is unstable and undergoes unfolding. Again, unfolding induces localized increases in curvature resulting in large peaks (*к* → 0.3 Å^-1^) for sections of the molecule length. Once sufficient unfolding occurs, the structure approaches a homogeneous, unfolded state (*κ* ≈ 0.06 Å^-1^).

To confirm, the *local* curvature is plotted as a function of time across the length of the carbyne molecule (Figure 7). Due to thermal fluctuations, the unfolding trajectory is highly stochastic, and the curvature plots are representative only. Both *n* = 72 and *n* = 144 are plotted as examples and are the same trajectories as the average curvatures plotted in Figure 6. For *n* = 72, a relatively low temperature is required for a stable three-loop structure (*T* = 50 K). Curvature is approximately constant (*κ* ≈ 0.27 Å^-1^, for a radius of approximately 3.7 Å) with slight variation along the molecular length due to temperature-induced oscillations. The two  peaks’ (*κ* ≈ 0.3 to 0.04 Å^-1^) occur approximately at the crossover of the carbon chains (see Figure 1c), necessitating a slight increase in local curvature. At a higher temperature (*T* = 200 K), there is enough energy to initiate unfolding. While globally the average radius increases, local unfolding induces increases in curvature in adjacent sections of the loop. Large peaks in the local curvature exceed 0.5 Å^-1^ before the structure  relaxes’ to a homogeneous, unfolded state (*κ* ≈ 0.12 Å^-1^). While limited in length, these increases in local curvature considerably impede unfolding at a low temperature and enhance stability in spite of the relatively high bending energy of the looped structure. For *n* = 144, again, low temperature results in a stable three-loop structure but at a higher range than *n* = 72 (*T* = 300 K, depicted). The thermal fluctuations and longer molecular length result in less prominent peaks as the effect of the crossover of the carbon chains is decreased. At a stable temperature, the curvature is relatively constant throughout the simulation (*κ* ≈ 0.11 Å^-1^, for a radius of approximately 9.0 Å). Increasing the temperature to induce unfolding again results in local increases in curvature to isolated sections of the molecule (exceeding 0.3 Å^-1^) while the *average* curvature decreases. Again, it is stressed that the peaks depicted in Figure 7 are stochastic and should be considered as representative only. However, all unfolded systems demonstrated significant increases in local curvature.

### Critical unfolding temperatures

While the specific increases in curvature are non-deterministic, a simple model can be formulated to determine the critical unfolding temperature. To theoretically explore the stability of the folded carbon (or carbyne) loops, first the stored bending strain energy, *U*_b_, in the system is defined, where [70]

(3) $$U_{b}=\frac{1}{2}D\;\int_{0}^{L}\kappa^{2}d\hat{s}=\frac{1}{2}\text{DL}\kappa^{2}$$

where *к* denotes the initial imposed curvature of the carbyne chain of length *L*. During unfolding, it is assumed that there is a decrease in bending energy over portion of the length, *αL*, where *α <* 1.0, due to a decrease in curvature from *к* to *βк*, where *β <* 1.0. Thus, the amassed change in energy due this unfolding across the molecular length can be formulated as

(4a) $$\Delta U_{b}=\frac{1}{2}D\int_{0}^{\mathrm{\alpha L}}\kappa^{2}d\hat{s}\;-\frac{1}{2}D\int_{0}^{\mathrm{\alpha L}}{\left(\beta \kappa \right)}^{2}d\hat{s}=\frac{1}{2}\mathrm{DL}\kappa^{2}\left(\alpha \left(1-\beta^{2}\right)\right)$$

Comparing to Equation 3, the change in energy due to local unfolding is a fraction of the total bending energy, as must be the case. The term *α*(1 *- β*^2^) < 1 by definition, where *α* captures the length of the chain unfolding and *β* is the decrease in curvature. In consideration of the simulation results, this is a stochastic term which cannot be calculated. We note that, due to thermal fluctuations, the curvature profile of the rings are constantly changing; calculating the bending strain energy for a particular case may result in a more accurate description for a single instance. Thus, we consider limiting cases only. The maximal case can be determined considering the upper bound of *α =* 1, wherein the entire loop may unfurl, and the minimum *β*. In the three-loop configuration, *κ* = 6*π/L*, while completely unfolded, *κ* = 2*π/L*, such that, for this particular structure, the lower bound of *β* is 1/3. With these two assumptions,

(4b) $$\Delta U_{b}=\frac{1}{2}\mathrm{DL}\kappa^{2}\left(\frac{8}{9}\right)=\frac{4}{9}\mathrm{DL}\kappa^{2}$$

Moreover, noting again that *κ* = 6*π/L*,

(4c) $$\Delta U_{b}=\frac{16\pi^{2}D}{L}$$

Note that here *D* represents the effective bending stiffness. We also presume that carbyne behaves as a flexible molecular chain with a temperature-dependent flexibility and finite rigidity at zero temperature. A common property of molecular chains in polymer science is the persistence length, *P*, defined as the characteristic length over which direction can be correlated [71], related to both temperature (*T*) and bending rigidity (*D*). For flexible molecules, the persistence length can be approximated as *P = D/k*_B_*T*, where *k*_B_ is the Boltzmann constant [72]. In a similar manner, persistence length is formulated here as a proxy for rigidity, assuming some finite persistence independent of temperature. As a consequence, the bending stiffness, *D*, can be directly represented as a function of temperature:

(5) $$D=P_{0}k_{B}T+D_{0}$$

where *P*_0_ is considered the temperature-independent persistence length. In effect, the apparent bending rigidity increases with temperature, also supported by previous theoretical results; a recent *ab initio* (temperature-free) investigation reports the bending stiffness to be in the order of 5.3(10^-2^) nN-nm^2^[23], while a finite temperature (300 K) molecular dynamics study reports a stiffness of approximately 13 to 20(10^-2^) nN-nm^2^[21]. Here, *D*_0_ is the rigidity at zero temperature (as carbyne is not ideally flexible) and thus is approximated as 5.3(10^-2^) nN-nm^2^.

At the critical condition for unfolding, the gained strain energy (Equation 4) must be sufficient to overcome a local energy barrier, *Ω*, where *Ω* is a combination of adhesion energy and required strain energy to unfold (e.g., local increase in curvature as depicted in Figure 7 and torsional and adhesion contributions) such that Δ*U*_b_ = *Ω*. Substituting (4) into (3c) and rearranging to solve for temperature results in

(6) $$T_{\text{unfolding}}=\left(\frac{\Omega }{16\pi^{2}P_{0}k_{B}}\right)L-\left(\frac{D_{0}}{P_{0}k_{B}}\right)$$

**Figure 8:**
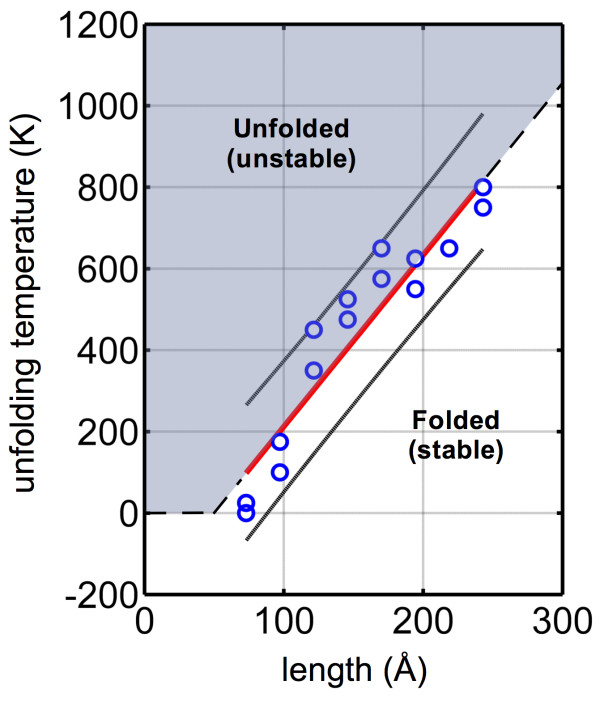
**Critical unfolding temperature (*T***_**unfolding**_**) versus molecule length.** Due to the stochastic nature of unfolding, simulation results indicate a range of possible unfolding temperatures for each length of carbyne model. The maximum and minimum of each length are plotted. For example, for *n* = 126 (*L* ≅ 170 Å), both unfolding and stable structures were observed at temperatures from 575 to 650 K (points plotted), but all simulations above 650 K unfolded, and all below 575 K remained stable. The results were fitted with a linear regression (red line), resulting in a temperature intercept of -204 ± 142 K and a slope of 4.2 ± 0.85 K Å^-1^ (with an associated *R*^2^ value of 0.889). The results can be associated with Equation 6. The regression can be used to delineate the folded (stable) and unfolded (unstable) states in an effective phase diagram. The 90% confidence intervals are also plotted, encapsulating all observed data points.

Using Equation 6 with the simulation results, the approximate unfolding temperature, *T*_unfolding_, can be predicted. The key assumption is that the unfolding process does not imply a constant decrease in energy (i.e., release of bending strain energy), and thus some energetic input, *Ω*, is required to allow deviation from the high-energy folded or looped state, which can be considered a temperature-dependent state of quasi-equilibrium. The maximum and minimum observed unfolding temperatures of each molecular length are plotted and fitted by linear regression (Figure 8). From the fitted parameters and assuming *D*_0_ ≅ 5.3(10^-2^) nN-nm^2^, both *P*_0_ and *Ω* can be calculated. From the temperature intercept (-204 ± 142 K), *P*_0_ is estimated as 110 to 610 Å (best fit with *P*_0_ = 187 Å). Note that this is not considered the persistence length of carbyne but only a temperature-independent contribution (such that carbyne will display finite persistence even at high temperatures) and thus a lower bound. As a comparison, the persistence length of DNA is similarly in the order of tens of nanometers [73,74]. Using the best fit value of *P*_0_ and Equation 5, the increase in stiffness for finite temperatures can be calculated. A temperature of 300 K results in a bending stiffness of 13.0 nN-nm^2^, in good agreement with previous computational results [21].

Using the fitted slope of 4.2 ± 0.85 K Å^-1^, the energy barrier to unfolding, *Ω*, is determined to be approximately 98 to 366 kcal mol^-1^ (best fit with *Ω* = 139 kcal mol^-1^), which agrees well with the magnitude of measured energy barriers (40 to 400 kcal mol^-1^). This range may be seemingly large as the energy of cohesion for the chains is in the order of 7 eV or approximately 160 kcal mol^-1^; one may expect the chains to break before unfolding. However, the barrier is due to the bending strain energy required, which, by definition, requires the involvement of numerous atoms (rather than a single cleavage site [75], for example). In consideration of the relatively large flexural rigidity of carbyne, such bending energy barriers can be quite significant. If we consider the change in curvature for *n* = 72, from approximately 0.27 Å^-1^ to local peaks of 0.5 Å^-1^, then we can approximate the length that undergoes the local increase in curvature by equating the energy barrier, *Ω*, with the local bending strain energy. For *n* = 72 at 200 K (for a bending rigidity of *D*_200*K*_ *=* 10.4 nN-nm^2^ by Equation 5), this results in local curvature increase in approximately 7.4 to 27.5 Å of the loop. This range of length is in good agreement with the size/span of the peaks depicted in Figure 8. Similarly, considering *n* = 144 at 725 K (for a bending rigidity of *D*_725*K*_ = 24.0 nN-nm^2^), with curvature increases from 0.11 Å^-1^ to local peaks of 0.3 Å^-1^, results in local curvature increasing in approximately 7.2 Å to 27.2 Å to develop the determined energy barrier, again in good agreement with Figure 8, which indicated multiple (but short spanning) peaks across the molecular length. It is noted that there is an intrinsic relationship between the magnitude of local curvature and necessary length, i.e., a longer length can develop the equivalent energy barrier with a smaller curvature as *U*_b_ ∝ *Lк*^2^.

## Conclusions

The results confirm that, while global unfolding implies an overall reduction in curvature, continuity of the molecular loop results in local increases in curvature, resulting in a small yet finite energy barrier to surpass. For longer loops (with less stored bending strain energy due to a decrease in curvature), a higher temperature (e.g*.*, kinetic energy) is required to induce unfolding. In contrast, short loops (with high bending energies) unfold at relatively low temperatures. Using carbyne as a platform, the potential for folding can serve to extend the accessible design space of such materials. It is noted that the heterogeneous/local curvature as depicted in the snapshots in Figure 3, as well as plotted in Figure 7, was not explicitly considered in terms of energy contribution. Rather, the limiting cases - the curvature of the three-loop structure and the curvature of an unfolded ring - were used to estimate the necessary energy. Here, all structures begin in an ideal configuration, and the deviations from the ideal curvatures are due to thermal fluctuations; the thermal energy (essentially molecular kinetic energy) must impose overcurvature to trigger the unfolding process. Since the heterogeneous curvatures are stochastic (the results plotted are only representative), temperature is used as a proxy to evaluate the necessary energy to unfold.

It behooves us to note that the looped carbyne structure modeled herein is not attainable experimentally and is intended as an ideal model platform to explore the unfolding phenomena. A similar idealized  bead-spring-type’ model could have been constructed but would be subject to the arbitrariness of parameterization. Carbyne provides a compromise - an ideal structure with physical, fundamental, and proven molecular-scale parameterization/behavior through the ReaxFF potential. It is the simplest case from a molecular perspective (a non-reactive homogeneous chain, no solvent, etc.) and is necessary to isolate and observe the thermal contribution to unfolding as well as the local curvature effect. Indeed, understanding the stability and mechanics of folded carbyne loops can be of use in modifying transport properties or triggering mechanisms in active molecular systems.

Finally, understanding the mechanical principles of such geometries will help not only in dissecting existing looped systems but also in designing and constructing commercially useful self-assembling nanostructures. For example, the local curvature increase may be isolated in a particular, flexible molecular  hinge’ or activated by an enzyme in biological systems. When one thinks of folding/unfolding at the molecular scale, DNA and similarly protein structures are likely to come to mind. In terms of insights to such structures, the governing folding/unfolding phenomenon is quite different from carbyne loops. However, there are insights even from this simple system; DNA can exhibit looped configurations, which can serve to suppress the formation of gene products, or facilitate compaction of DNA as a whole [26-31,76,77]. The size of the loops also affects the mechanical stability [26-28] and has been analyzed via elastic assumptions [29] and thermodynamic cost [30]. Similar to the carbyne system here, larger loops are shown to be more stable. The observation that local curvature undergoes an increase may shed light into the attainment of such structures. Indeed, for small DNA looped structures to be stable, extensive local curvature is required (which can be potentially controlled by sequence; see [77] and references therein). While at a different scale, clearly there is an interplay between curvature, local flexibility, and temperature similar to that of the structures observed here. There are no *direct* insights from carbyne to macromolecules such as DNA, just as the general study of overcurvature in collapsible laundry baskets was not applied at the molecular scale here. But there are indeed potential *indirect* corollaries.

While carbon chains have been primarily studied as extensions from graphene [78] or carbon nanotubes [79,80], isolated carbynes and related structures may inspire an even smaller generation of nanomaterials, with increased functionality due to their intrinsic flexibility and ability to attain exotic topologies. Development of looped systems may lead to novel devices that  unfold’ per design with some external event - a potential novel nanoscale trigger - motivated by commercial pop-up tents and collapsible laundry hampers.

## Competing interests

The author declares no competing interests.
